# Another Case of Lisinopril-Induced Acute Pancreatitis

**DOI:** 10.7759/cureus.19488

**Published:** 2021-11-11

**Authors:** Jeffrey Baum, Aaron Walfish, Moshe Fenster, Yair Miller, Mindy Kresch

**Affiliations:** 1 Surgery, New York Medical College, New York, USA; 2 Gastroenterology and Hepatology, Icahn School of Medicine at Mount Sinai, Elmhurst Hospital Center, Elmhurst, USA; 3 Internal Medicine, Westchester Medical Center, New York, USA; 4 Anesthesia, New York Medical College, New York, USA; 5 Dermatology, New York Medical College, New York, USA

**Keywords:** side effects of lisinopril, angiotensin-converting enzyme inhibitors, acute pancreatitis, drug-induced pancreatitis, lisinopril

## Abstract

Lisinopril as a cause for acute drug-induced pancreatitis is an emerging phenomenon that due to its generally low-risk profile often goes unnoticed. The true incidence of drug-induced pancreatitis is unknown, probably because of its nonrecognition among differential diagnosis. Only a handful of lisinopril-induced pancreatitis has been discussed in the literature, and little epidemiological evidence exists to establish true causality. Additionally, many of these reports have been met with skepticism claiming that it is difficult to isolate a true cause since many of these patients had comorbidities or were concomitantly taking other medications that may have contributed to the pancreatitis. Here, we report a case in which a generally otherwise healthy patient presented with acute drug-induced pancreatitis caused by an angiotensin-converting enzyme (ACE) inhibitor taken eight weeks prior to the onset of symptoms. The drug was immediately stopped, and the patient recovered well, with no complications.

## Introduction

Angiotensin-converting enzyme (ACE) inhibitors were introduced three decades ago and have since become the mainstay for treatment of diseases such as hypertension, congestive heart failure, postmyocardial infarction, diabetic nephropathy, and chronic kidney failure with proteinuria [1]. They are generally safe and have excellent tolerability with a low risk for adverse effects. Drug-induced acute pancreatitis (AP) is a well-documented, albeit rare occurrence that has been discussed extensively in the literature [2]. ACE inhibitors do indeed appear on the list of known drugs to cause AP, although in exceedingly reduced numbers [3,4]. The handful of case reports that implicate lisinopril in drug-induced pancreatitis was typically seen in patients who either had other comorbidities or were taking other medications that may have contributed to the AP. Here, we present a generally otherwise healthy male who presented with AP determined to be caused by lisinopril.

## Case presentation

**Figure 1:**
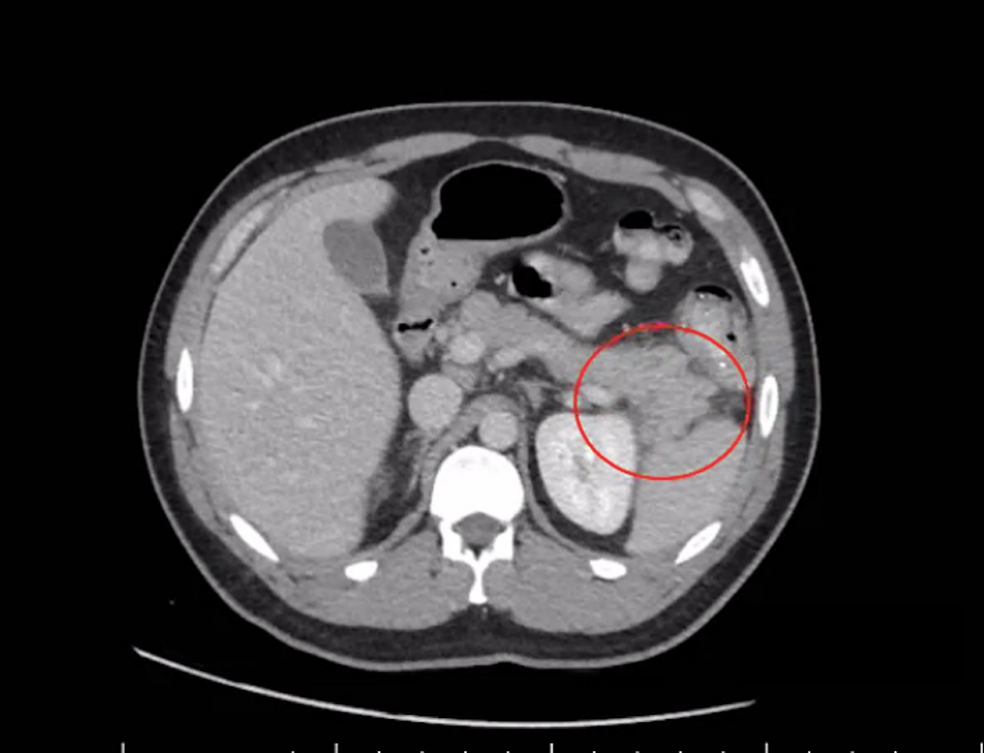
APCT scan with contrast showing mild pancreatitis (red circle) involving the pancreatic tail APCT: abdomen/pelvis computed tomography.

A 34-year-old male, with a past medical history of stage one hypertension, presented to the emergency room with a two-day history of acute onset, severe, constant, epigastric abdominal pain that radiated to the back with associated nausea and vomiting. His vital signs were within normal limits, and laboratory values were significant for elevated lipase levels of 123 (reference range: 13-60 U/L), alanine transaminase of 55 (reference range: 0-41 U/L), and aspartate aminotransferase of 42 (reference range: 5-40 U/L). All other laboratory values were within normal limits including hematologic, electrolyte, lipid panel, kidney function, and urine analysis. Abdomen/pelvis computed tomography (APCT) scan with contrast agent showed mild pancreatitis involving the pancreatic tail with fat stranding and trace free fluid (Figure 1). 

**Figure 2:**
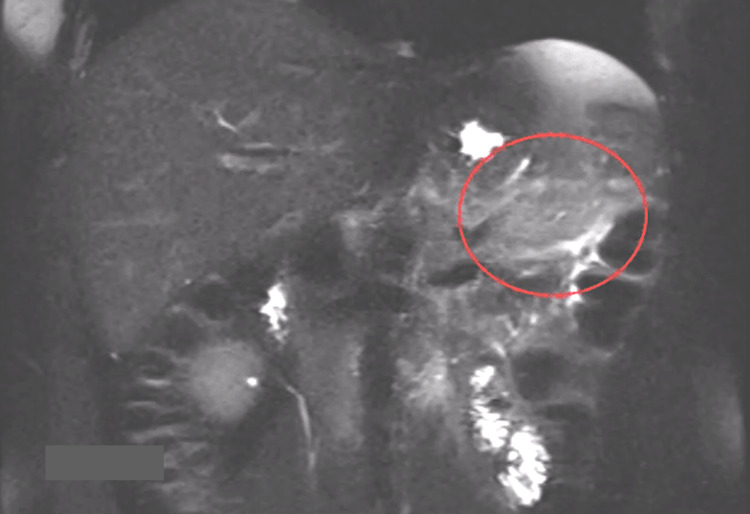
Coronal T2-weighted with fat-sat showing peripancreatic edema (red circle) Fat-sat: fat-saturation.

**Figure 3:**
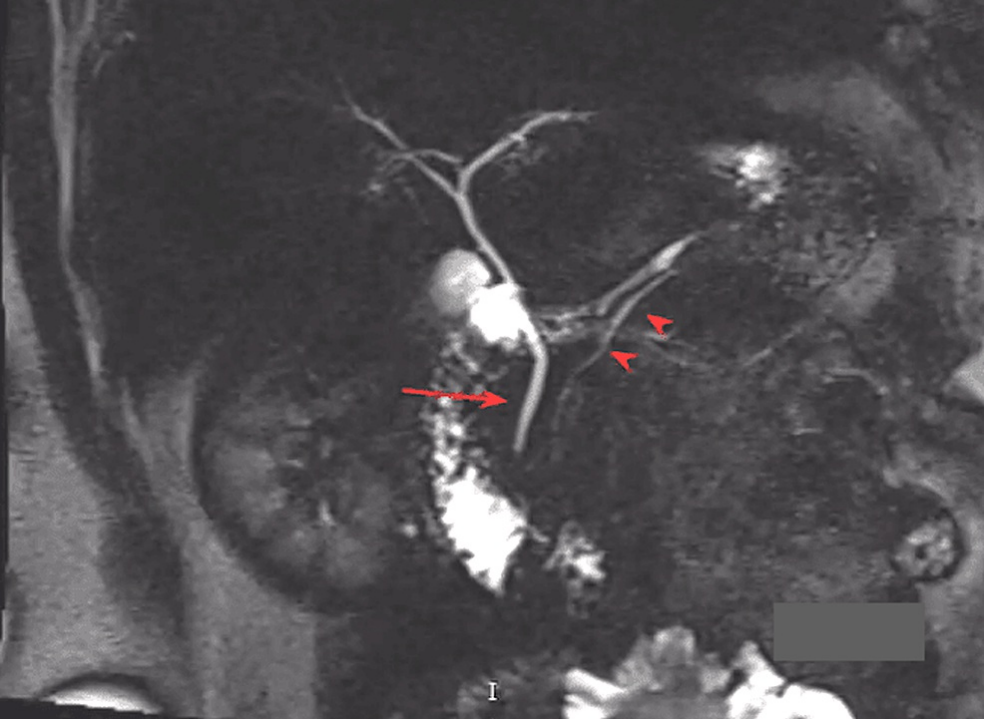
Thick Slab MRCP showing normal caliber common bile duct (red arrow) and pancreatic duct (red arrowheads) MRCP: magnetic resonance cholangiopancreatography.

The colon demonstrated mild focal wall thickening in the region of the splenic flexure adjacent to the inflammatory changes of the pancreas. Magnetic resonance cholangiopancreatography (MRCP) confirmed peripancreatic edema and fat stranding (Figure 2) and showed normal caliber common bile duct and pancreatic duct (Figure 3), making gallstone pancreatitis a highly unlikely diagnosis.

 

About eight weeks prior to the admission, he had started a 5 mg per day regimen of lisinopril for stage one hypertension. Three weeks later, his dosage was increased to 10 mg per day to better reach therapeutic goals. Only another medication, duloxetine, 30 mg per day, was contemporary taken for anxiety, for the past 10 years. He was not taking any other medications or supplements. An extensive workup was completed to determine the etiology of the pancreatitis. Gallstone pancreatitis was effectively ruled out, he denied any alcohol abuse, imaging excluded congenital anomalies and revealed normal pancreatic and bile duct caliber, calcium and triglyceride levels were within normal limits, and antinuclear antibodies and anti-DNA double-stranded antibodies were negative (although immunoglobulin G4 was not performed, at that time). All other known causes of acute pancreatitis were subsequently ruled out, leading to the exclusionary diagnosis of drug-induced AP. The lisinopril was promptly discontinued and switched to amlodipine 10 mg per day. The patient was treated with fluids and recovered well, with no complications. At one-month and one-year follow-up, there were no recurrences of AP. 

## Discussion

The etiology of AP in developed countries is most commonly due to gallstone obstruction (38%) and alcohol abuse (36%) along with uncommon but well-established causes such as hypertriglyceridemia, infections, autoimmune conditions and hypercalcemia, among others [2]. Another rare cause for AP is drug induced and has been estimated to occur with an incidence of 0.1%-2% [3-4]. Although a link between many commonly prescribed drugs and AP has been discussed extensively in the literature, the exact mechanism of injury to the pancreas has not yet been completely elucidated but may be a result of pancreatic duct obstruction secondary to angioedema from increased bradykinins as well as autodigestion from prematurely activated pancreatic enzymes [5]. Management of drug-induced pancreatitis includes supportive care and immediate withdrawal of the offending medication. Failure to identify a drug as the causal agent can worsen the disease course or lead to recurrent pancreatitis.

Of the common drugs associated with AP, ACE inhibitors have an even lower incidence of causality [5-11]. They are generally safe and have excellent tolerability with a low risk for adverse effects. The most common side effects of ACE inhibitors include dry cough, angioedema, first-dose hypotension, dizziness, hyperkalemia, and renal dysfunction [12-14].

There are only a handful of case reports that single out ACE inhibitors as a cause for drug-induced pancreatitis, and little epidemiological evidence exists to establish true causality [15,16]. Furthermore, the accuracy of many of these reports has been met with skepticism claiming that it is difficult to isolate a true cause since many of the patients in these reports showed significant comorbidities or were concomitantly taking other medications that may have contributed to the acute pancreatitis. Indeed, in a systematic review of case reports, it was determined that scant evidence exists to prove a firm inciting relationship between drugs and pancreatitis due to unclear etiologies of the pancreatitis [17-20].

Our case report is important as it showcases a generally otherwise healthy patient at very low risk for pancreatitis, with no significant past medical history who presented with acute onset pancreatitis subsequently determined to be drug induced from an ACE inhibitor. He had no history of alcohol abuse, MRCP showed no evidence of gallstone pancreatitis, and all other causes of AP were ruled out. After discontinuing lisinopril, there were no reports of any recurring AP during a one-year follow-up. Additionally, our patient scored a 7 on the Naranjo Adverse Drug Reaction Probability Scale which indicates a high probable causal link between the drug and the AP. Importantly, a definitive link can only truly be confirmed after a drug reexposure [16]; however, in our case, a lisinopril rechallenge was deemed inappropriate and unethical to attempt. This case report fortifies the importance of considering lisinopril as a possible etiological agent. Prompt cessation of the drug is warranted to reduce the severity and recurrence of the disease.

## Conclusions

In conclusion, this case report demonstrates further evidence of lisinopril-induced acute pancreatitis in an otherwise generally healthy male and adds to the growing pile of literature that implicates antihypertensives in causing AP. While there have been previous reports of ACE inhibitors causing acute pancreatitis, many of these patients either had other comorbidities or were taking other medications that may have contributed to the AP.

The significance of this case report highlights the importance of conducting a thorough review of every patient’s complete medication list when exploring causes of AP. The ruling out of other causes of acute pancreatitis such as gallstone pancreatitis, alcoholism, hypertriglyceridemia, hypercalcemia, autoimmune conditions and infections, among others, should alert clinicians to consider even the fairly safe drugs as a possible causal agent. Swift withdrawal of the offending medication should then be immediately initiated and replaced with an acceptable alternative. 
